# Maintenance of the human memory T cell repertoire by subset and tissue site

**DOI:** 10.1186/s13073-021-00918-7

**Published:** 2021-06-14

**Authors:** Michelle Miron, Wenzhao Meng, Aaron M. Rosenfeld, Shirit Dvorkin, Maya Meimei Li Poon, Nora Lam, Brahma V. Kumar, Yoram Louzoun, Eline T. Luning Prak, Donna L. Farber

**Affiliations:** 1 Department of Microbiology and Immunology, Columbia University, New York, NY USA; 2 Columbia Center for Translational Immunology, Columbia University, New York, NY USA; 3 Department of Pathology and Laboratory Medicine, Perelman School of Medicine, University of Pennsylvania, Philadelphia, PA USA; 4 Department of Mathematics, Bar Ilan University, Ramat Gan, Israel; 5 Department of Pathology and Cell Biology, Columbia University, New York, NY USA; 6 Department of Surgery, Columbia University, New York, NY USA

**Keywords:** Immunogenomics, Immunology, T cell, Immunity

## Abstract

**Background:**

Immune-mediated protection is mediated by T cells expressing pathogen-specific T cell antigen receptors (TCR) that are maintained at diverse sites of infection as tissue-resident memory T cells (TRM) or that disseminate as circulating effector-memory (TEM), central memory (TCM), or terminal effector (TEMRA) subsets in blood and tissues. The relationship between circulating and tissue resident T cell subsets in humans remains elusive, and is important for promoting site-specific protective immunity.

**Methods:**

We analyzed the TCR repertoire of the major memory CD4^+^ and CD8^+^T cell subsets (TEM, TCM, TEMRA, and TRM) isolated from blood and/or lymphoid organs (spleen, lymph nodes, bone marrow) and lungs of nine organ donors, and blood of three living individuals spanning five decades of life. High-throughput sequencing of the variable (V) portion of individual TCR genes for each subset, tissue, and individual were analyzed for clonal diversity, expansion and overlap between lineage, T cell subsets, and anatomic sites. TCR repertoires were further analyzed for *TRBV* gene usage and CDR3 edit distance.

**Results:**

Across blood, lymphoid organs, and lungs, human memory, and effector CD8^+^T cells exhibit greater clonal expansion and distinct *TRBV* usage compared to CD4^+^T cell subsets. Extensive sharing of clones between tissues was observed for CD8^+^T cells; large clones specific to TEMRA cells were present in all sites, while TEM cells contained clones shared between sites and with TRM. For CD4^+^T cells, TEM clones exhibited the most sharing between sites, followed by TRM, while TCM clones were diverse with minimal sharing between sites and subsets. Within sites, TRM clones exhibited tissue-specific expansions, and maintained clonal diversity with age, compared to age-associated clonal expansions in circulating memory subsets. Edit distance analysis revealed tissue-specific biases in clonal similarity.

**Conclusions:**

Our results show that the human memory T cell repertoire comprises clones which persist across sites and subsets, along with clones that are more restricted to certain subsets and/or tissue sites. We also provide evidence that the tissue plays a key role in maintaining memory T cells over age, bolstering the rationale for site-specific targeting of memory reservoirs in vaccines and immunotherapies.

**Supplementary Information:**

The online version contains supplementary material available at 10.1186/s13073-021-00918-7.

## Background

Adaptive immune responses mediated by T lymphocytes are critical for protection against diverse pathogens and depend on the rapid mobilization of T cells to infection sites [1]. In primary responses, antigen-specific naïve T cells in lymphoid tissue become activated, clonally expand, and differentiate into effector cells which migrate to tissue sites for immune-mediated pathogen clearance. A subset of these previously activated T cells persists as heterogeneous memory T cell subsets which either become retained in tissues as non-circulating tissue-resident memory T cells (TRM) [1, 2], or circulate through blood and tissue sites as central-memory (TCM), effector-memory (TEM), and terminally differentiated effector cells (TEMRA) subsets [3]. TRM are phenotypically and functionally distinct from circulating memory subsets, mediate optimal protective immunity to site-specific pathogens compared to circulating subsets, and are implicated in anti-tumor immunity [4–7]. The role of circulating memory subsets in protection and their relationship to TRM remains enigmatic. Understanding how memory T cells circulating in blood relate to memory T cell subsets in the tissues is of central importance for defining and monitoring protective T cell responses in humans.

Each T cell expresses a unique T cell antigen receptor (TCR) comprised of α and β chains encoded by *TRA* and *TRB* genes which derive from the rearrangement of *TRAV* and *TRAJ*, or *TRBV*, *TRBD*, *TRBJ* gene segments, respectively. These gene rearrangements can give rise to a theoretical diversity of 10^15^ distinct β-chains [8, 9]. High-throughput sequencing (HTS) of the variable regions of TCR genes has enabled quantitative assessments of clonal diversity and expansion within and between individuals, in health and in disease [10, 11]. By HTS, the measured TCR diversity for human naïve T cells is 10^7^–10^8^ different clones; memory T cells exhibit extensive clonal expansion and sigificantly lower diversity, particularly for CD8^+^T cells [12–14]. TCR clonal analysis has also be used to identify tumor-associated T cell clonal expansions for tracking in peripheral blood [15] and specific clonal expansions within subsets that are associated with disease states [16, 17]. However, a comprehensive baseline assessment of the distribution of clonally expanded memory T cells across subsets and tissues has not been accomplished and is necessary to interpret their significance in disease states.

Here, we investigated how the TCR repertoire of memory T cells is distributed by subset and location by TCR sequencing of the major CD4^+^ and CD8^+^ memory subsets isolated from blood, lymphoid tissues, and lungs of individual organ donors using a tissue resource we have extensively validated for human immune cell studies [13, 18–20]. Quantitative and qualitative aspects of the TCR repertoire were analyzed as a function of the memory T cell subset, tissue, lineage (CD4 vs. CD8), and individual. We found that clonal diversity and expansion were intrinsic features of the lineage and subset, with CD8^+^TEMRA cells having the highest clonal expansion, CD4^+^TCM cells the lowest, and TRM and TEM at intermediate diversity independent of the tissue of origin. Accordingly, the extent of overlap between sites was highest for TEMRA and lowest for TCM cells, while TRM and TEM exhibited significant clonal overlap suggesting a common origin. We also observed tissue-specific expansions for memory clones and that qualitatively similar clones segregated more by tissue, than by subset. Finally, we detected a loss of diversity of circulating but not TRM subsets with age. Together, these results indicate that while memory T cells are maintained as highly expanded clones across the body, tissues can serve as reservoirs for maintaining memory T cell diversity and tissue-adapted T cell specificities.

## Methods

### Acquisition of human tissue

Human tissues were obtained from deceased organ donors at the time of organ acquisition for clinical transplantation through an approved protocol with LiveOnNY, the organ procurement organization for the New York metropolitan area [21]. We obtained blood, multiple lymphoid sites (bone marrow (BM), lymph nodes (LN), spleen (Spl)), and lungs from human organ donors. Donors were free of cancer and negative for hepatitis B, C, and HIV. Isolation of tissues from organ donors does not qualify as “human subjects” research, as confirmed by the Columbia University IRB. For isolation of blood from living individuals, blood was drawn via venipuncture from consented volunteers, as approved by the Columbia University IRB. The individual donors in this study represented a diverse population spanning five decades of adult life (29–63 years) (Table 1).

**Table 1:** Characteristics of organ and blood donors in this study

Donor ID #	Age	Sex	C^a^	E^b^	HLA type^c^								
					A	B	BW4/BW6	C	DR	DR51/52/53	DQB1	DQA1	DPB1
383	39	M	−	+	1/2	8/44	+/+	07/16	13/17	−/52/−	2/6	01/05	02:01/14:01
324	56	M	+	+	2/32	13/35	+/+	4/6	7/12	−/+/+	2/7	02/05	04:02/10:01
299	29	M	+	+	23/24	48/49	+/+	7/8	4/11	−/+/+	8/6	01/03	04:02/14:01
255	63	F	+	+	24	61	−/+	10	4	−/−/+	8	03:01	04:02
233	26	F	−	+	2	35/62	−/+	9/15	13/16	+/+/−	DQ6/DQ7	n/a	n/a
287	34	M	+	+	2/68	18/53	+/+	4/12	13/15	+/+/−	6/6	01/01	04:01/104:01
280	26	M	−	+	11	35/44	+/+	5/12	4/13	−/+/+	6/7	01:03/03:03	02:01/03:01
229	32	M	−	+	2/30	13/27	+/−	2/6	11/17	−/+/−	DQ2/DQ7	n/a	n/a
466	59	M	−	−	1/2	8/44	+/+	05/07	4/4	−/−/53	7/8	03:01/03:03	06:01/104:01
Blood 1	55	F	+	+	n/a	n/a	n/a	n/a	n/a	n/a	n/a	n/a	n/a
Blood 2	32	F	n/a	n/a	n/a	n/a	n/a	n/a	n/a	n/a	n/a	n/a	n/a
Blood 3	29	M	n/a	n/a	n/a	n/a	n/a	n/a	n/a	n/a	n/a	n/a	n/a

^a^Serostatus for cytomegalovirus (C) (+ or neg (−))

^b^Serostatus for Epstein-Barr virus (E)

^c^HLA typing for class I and class II. n/a, not measured

### Isolation of mononuclear cells from human tissues

Tissue samples were maintained in cold saline and brought to the laboratory within 2–4 h of procurement. Spleen, lung, and LN draining the lung were processed using enzymatic and mechanical digestion as previously described resulting in high yields of live leukocyte s[13, 18, 21]. Mononuclear cells were isolated from blood and BM with Lymphocyte Separation Medium (Corning, USA).

### Purification of memory T cells

For isolation of memory subsets by fluorescence-activated cell sorting, single cell suspensions were stained with fluorochrome conjugated antibodies in sorting buffer (PBS/1% fetal bovine serum). Stained cells were sorted using the BD Influx high-speed cell sorter (BD Biosciences). CD4^+^ and CD8^+^ T cells were fractionated into four distinct subsets: TCM (CD45RA^-^ CCR7^+^), TEM (CD45RA^-^ CCR7^-^CD69^-^), TRM (CD45RA^-^ CCR7^-^ CD69^+^), and TEMRA (CD45RA^+^ CCR7^-^) cells. TEM and TRM were isolated for both CD4^+^ and CD8^+^ T cells; however, only CD8^+^TEMRA and CD4^+^TCM were isolated for analysis, due to low frequencies of CD4^+^TEMRA and CD8^+^TCM in blood and tissues as previously reported [13]. The complete gating strategy is presented in Additional file 1: Fig. S1.

### DNA extraction

Sorted T cells were pelleted and resuspended in cell lysis solution (Qiagen) and DNA was isolated from cell lysate using the Gentra Puregene kit (Qiagen) for 9 donors (D383, D466, D324, D299, D255, D233, HD1, HD2, and HD3). For 3 donors (D287, D280, and D229), DNA and RNA were extracted using an RNA/DNA kit (AllPrep DNA/RNA mini kit, Qiagen). Upon extraction of DNA from purified T cells, DNA was divided into equal parts for replicate amplification and sequencing. The amount of DNA sequenced per sample was constant for the first two replicates of each individual donor, with the exception of blood from D383, in which fewer cells were obtained. The quantity of DNA isolated and sequenced per sample is indicated in Additional File 1: Table S1.

### TRB gene amplification, library preparation, and sequencing

Targeted PCR was used for amplification of *TRB* sequences from genomic DNA, using a cocktail of forward primers specific for framework region 2 (FR2) sequences of 23 *TRBV* subgroups (gene families), and 13 *TRBJ* region reverse primers adapted from the BIOMED2 primer series [22]. Amplicons were purified using the Agencourt AMPure XP beads system (Beckman Coulter, Inc.). Second-round PCRs to generate the sequencing libraries were carried out using NexteraXT Index Primers S5XX and N7XX. Libraries were sequenced using an Illumina MiSeq in the Human Immunology Core Facility at the University of Pennsylvania. 2 × 300 bp paired end kits were used for all experiments (Illumina MiSeq Reagent Kit v3, 600 cycle, Illumina Inc., Cat. No. MS-102-3003).

### TCR read counting and clone mapping

Raw reads were pre-processed using pRESTO [23] v0.5.10 and then annotated using IgBLAST’s igblastn command v1.17.0 [24] as shown in Additional File 1: Supplementary Methods. For IgBLAST, the IMGT human TRBV and TRBJ reference databases from October 24, 2019, were used. Low-quality sequences were removed if their average shred quality score was less than 30, stretches of bases on each end of all reads that were of low average quality were removed, short sequences (100 bases or fewer) were discarded, and individual bases with a phred low quality score of less than 30 were replaced with an N. Finally, any sequences with more than 10 such Ns were removed. IgBLAST was then run on the resulting filtered sequences producing AIRR-compliant output files (Additional File 1: Supplementary Methods).

AIRR-compliant output files were then imported into ImmuneDB v0.29.9 [25, 26] using the *immunedb_import* function [25, 26] (Additional File 1: Supplementary Methods). We defined clonally related sequences as those with identical *TRBV* and *TRBJ* gene segments and CDR3 amino acid sequences. We required that a unique sequence be detected at least twice (within an individual) in order to be designated a clone to reduce over estimation of clones due to sequencing errors.

### TCR diversity, clonality, and TRBV usage

TCR diversity and clonality analyses were performed using replicate one (see Additional File 1: Table S2) per subset per donor to normalize the cell input. The clonal summary plots were generated using the *clonal.proportion* function in the tcR package in R. To calculate clonality, given a clone, denoted x, frequency denoted p(x), and total set size of unique clones denoted L, $Clonality(X)=1-\frac{-{\sum}_{x\in X}p(x) lo{g}_2p(x)}{- lo{g}_2\frac{I}{L}}$. Here, clonality was calculated as normalized entropy as described [13, 27].

For Vβ analysis, we applied a copy number cut-off for clones below 50% of the mean copy number frequency of each sample and used replicate 1 for each sample. The 50% mean copy number cut-off was calculated as follows: the total number of copies in a sample was summed and then divided by the number of unique clones, to derive the mean copy number for that sample. The mean copy number was halved, and any clones with a copy number below the 50% mean copy number frequency were removed from the sample. We calculated the 50% mean copy number cut-off for each sample. We counted the number of unique clones with each *TRBV* gene call. For *TRBV* frequencies, we calculated the percentage of total clones that utilized each *TRVB* gene. For heatmap visualization, percentages were centered and scaled by column using the *pheatmap* R package so that red is a high percentage and blue is a low percentage. The heatmap columns were clustered using the complete linkage option in *pheatmap*. Data from all 12 donors were aggregated in the bar graph comparing CD4^+^ and CD8^+^T cells. Principal component analysis was visualized using the *factoextra* package in R.

### Cosine similarity

Cosine similarity analysis was performed on replicates 1 and 2 per sample to normalize cell input (Additional File 1: Table S2). We applied a copy number cut-off, removing clones that were below 50% of the mean copy number frequency in each sample, and calculated the cosine similarity using the *cosine* function in R. We show the mean of four pairwise comparisons between the two replicates per sample, reported in the heatmap. For bar plots of cosine values, cosine similarity was normalized to account for the between-subject variability and the standard error was calculated from the normalized data.

### Edit distance calculation and visualization

Overlappping clones and non-productive rearrangements were removed from the dataset, and, based on a set of 500 random clones per sample, we calculated the edit distance (Levenshtein distance represents the number of insertions, deletions, and substitutions required to change one sequence to the other [28]) between every pair of CDR3 amino acid sequences and the Kullback–Leibler (KL) divergence [29] between the *TRBV* gene composition of the samples. Edit distances were computed, aggregated, and graphed or visualized as tSNE plots (for 250 random clones) with jellyfish.levenshtein_distance, using the default settings. For the KL divergence (for 500 clones), we computed the frequency of each Vβ in each sample (p_i_(v) and p_j_(v) for samples i and j), and computed the symmetrized KL divergence:

$$ 1.D\left({p}_i\Big\Vert {p}_j\right)=\sum \limits_v{p}_i(v)\mathit{\ln}\frac{p_i(v)}{p_j(v)}+{p}_j(v)\mathit{\ln}\frac{p_j(v)}{p_i(v)} $$

A pseudo count of 1 was added to each v gene for the estimate of the frequencies.

For tSNE analysis, we combined all CDR3 sequences and reduced their dimensions to 2 using tSNE [30] with the default settings. Replicates 1 and 2 were used for this analysis.

### Statistical analysis

Descriptive statistics (means, SEM, median) were calculated for each cell subset and clonality and overlap (Cosine) were calculated in R. Significant differences in subset clonality were assessed using a Student’s t test.

## Results

### Isolation of circulating and tissue resident memory subsets from multiple sites

We obtained blood, multiple lymphoid sites (bone marrow (BM), lymph nodes (LN), spleen (Spl)), and lungs from human organ donors through a tissue resource and process we have extensively validated for immune cell studies [13, 18, 20, 31, 32]. The individual donors in this study represented a diverse population spanning five decades of adult life (29–63 years) (Table 1). Consistent with our previous results on phenotype and transcriptome profiling of T cell subsets in tissues [13, 21, 31, 33], the predominant tissue T cell subset is TEM (CD45RA^-^CCR7^-^) for both CD4^+^ and CD8^+^T cells, TRM are defined as CD69^+^TEM, CD4^+^T cells contain TCM (CD45RA^-^CCR7^+^), and CD8^+^T cells contain TEMRA cells (CD45RA^+^CCR7^-^) (Additional File 1: Fig. S1A, B). Naïve (CD45RA^+^CCR7^+^ ) T cells are present in lower frequencies in tissues compared to non-naïve subsets (and are negligible in spleen and lungs) and compared to blood which contains 40–60% naïve T cells for this age range (Additional File 1: Figure S1B and [13, 32]). For the sites studied here, lungs contained the highest TRM frequencies among total CD4^+^ and CD8^+^ T cells (60%), followed by LN (30%), spleen, and BM (10–20%), while blood lacked TRM (Additional File 1: Fig. S1A, B).

**Fig. 1:**
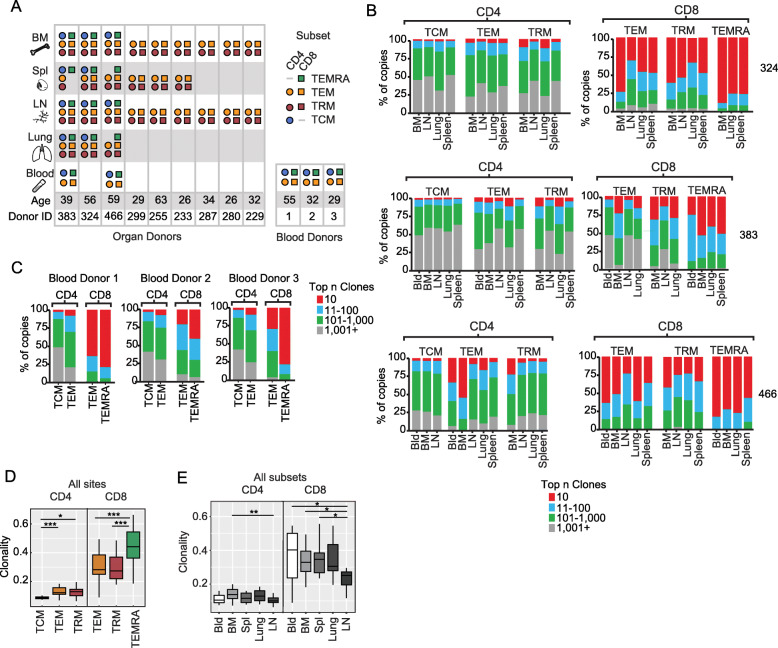
T cell receptor (TCR) diversity of memory cells is coupled to lineage and subset identity. **A** Tissue and subset origin of samples analyzed in this study derived from 12 individuals. T cell subsets included TEM (effector memory) and TRM (resident memory) for CD4^+^ and CD8^+^T cells, CD4^+^TCM (central memory), and CD8^+^TEMRA (terminally differentiated effector), isolated from blood, bone marrow (BM), spleen (Spl), lymph node (LN, lung-draining), and lungs of organ donors; TEM, TCM, and TEMRA cells were isolated from blood of three living donors. **B** Clonal expansion plots show proportion of top n clones per sample for CD4^+^ (left) and CD8^+^ (right) T cell subsets (TCM, TEM, TRM, and TEMRA) from indicated sites for three representative organ donors (donor number is given on the right). **C** Clonal expansion plots as in **B** for each of the three blood donors. The same clone rank legend (bar color scale) applies to both **B** and **C**. **D** TCR clonality was calculated (see methods) for each subset from all sites and donors in **A**. Boxplot depicts clonality by cell subset (all tissue sites combined) as 25% quantile (lower), median (middle), and 75% quantile (upper) and whiskers at minimum and maximum values. (CD4^+^: TCM, *n* = 15 TEM, *n* = 32; TRM, *n* = 27; CD8^+^: TEM, *n* = 31; TRM, *n* = 26; TEMRA, *n* = 14). **E** Clonality of T cells (calculated as in **D** stratified by tissue site (all subsets combined) (CD4^+^: Bld, *n* = 10, BM, *n* = 21; Spl, *n* = 14; Lung, *n* = 8; LN, *n* = 21; CD8^+^: Bld, *n*=10; BM, *n* = 21; Spl, *n* = 13; Lung, *n* = 9; LN, *n* = 18). Students t test. *P < 0.05, **P < 0.01, ***P < 0.001

As the goal of the study was to assess the clonal relatedness of previously primed (non-naïve) T cells between different tissues and blood by TCR gene sequencing, we sorted the major subsets of non-naïve CD4^+^ and CD8^+^T cells from the five sites. Thus, CD8^+^TEM, TRM, and TEMRA, and CD4^+^TEM, TRM, and TCM were sorted from blood and/or different tissue sites of 9 organ donors, and blood of 3 living individuals, resulting in 148 different biological samples (Fig. 1A). (CD8^+^TCM and CD4^+^TEMRA represented low frequency populations [13] and were not included in the analysis.) Total DNA was isolated from each purified cell subset and divided into two replicate samples from which the variable portion of the *TRB* gene containing V, D, and J segments and encoding the CDR3 region [11, 33] was PCR- amplified, used to generate libraries and sequenced (Additional File 1: Fig. S1, Table S1).

### T cell repertoire diversity and clonality is determined by lineage and subset

Individual *TRBV* and *TRBJ* gene segments, CDR3 sequences, and clone counts were identified from the resultant 84 million valid sequences in this dataset using ImmuneDB [25, 26] (see methods). Based on sequencing an equivalent number of sorted cells, we detected an average of 3750 clones per CD4^+^T cell sample and 1382 clones per CD8^+^T cell sample across all individuals (Additional File 1: Table S2), consistent with previous observations that human CD8^+^T cells are more clonally expanded than CD4^+^T cells [12, 13]. We then asked whether the TCR repertoire within specific subsets or tissues varied by the extent of diversity and/or clonal expansion. Calculating the percent of the TCR repertoire that was represented by the top most abundant clones (top 10, 100, 1000, > 1001) revealed that memory CD8^+^T cells contained highly abundant clones, such that the top 10 clones comprised up to 80% of the T cell repertoire, whereas for CD4^+^T cells, the top 10 clones comprised < 10% of the total T cell repertoire for all subsets and tissues (Fig. 1B, C).

The highly abundant clones among CD8^+^T cells for each donor were largely within the CD8^+^TEMRA subset in all sites, followed by CD8^+^TEM and TRM (Fig. 1B, C). For CD4^+^ subsets, TEM exhibited the highest clonal expansion that was greater than or comparable to TRM, while TCM exhibited the lowest clonal expansion (Fig. 1B, C). All individual donors exhibited a similar hierarchy of clonal expansion from highest to lowest: CD8^+^TEMRA > CD8^+^TEM and CD4^+^TRM > CD4^+^TCM (Fig. 1B), and subsets in the blood maintained the same hierarchy (TEMRA>TEM>TCM) (Fig. 1C). These clonal hierarchies and large clonal expansions were found for CMV-seropositive and CMV-seronegative donors (Fig. 1B, D, E). Clonal expansion was therefore intrinsic to lineage and subset.

As another quantitative measure of repertoire diversity that incorporates clonal expansion, we calculated TCR clonality, ranging from 0 (least clonally expanded; maximally diverse) to 1 (monoclonal, no diversity; see methods). Compiling T cell subset results from all sites (blood and tissues) revealed that clonality is a feature of the subset, ranked from highest to lowest CD8^+^TEMRA>CD8^+^TEM~TRM>>CD4^+^TEM~CD4^+^TRM>>CD4^+^TCM (Fig. 1D). Moreover, clonality did not differ significantly by tissue; rather, high clonality was a feature of the CD8^+^lineage, consistent with previous studies [12, 13]. One notable exception was that both CD8^+^ and CD4^+^T cells in LN exhibited a higher diversity and lower clonality than other sites (blood, BM, lung) (Fig. 1E). These results indicate that quantitative aspects of the memory T cell repertoire are determined by subset, independent of site of origin; however, LN maintained higher TCR diversity compared to other sites.

**Fig. 2:**
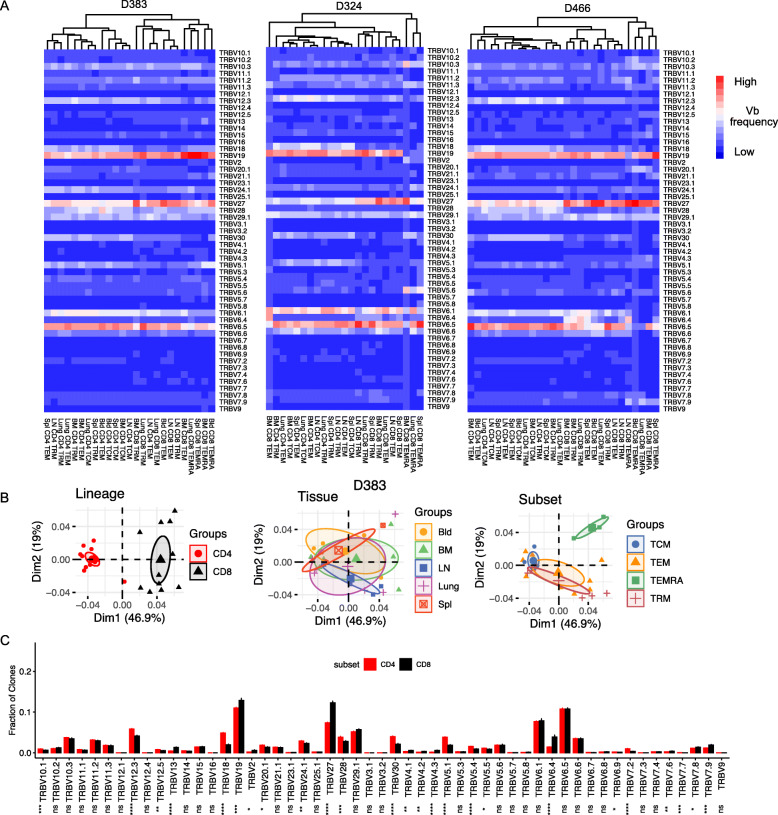
*TRBV* gene usage differs between CD4^+^ and CD8^+^T cell lineage and not by tissue or subset. **A** Heatmap of *TRB* gene usage of T cell clones from individual samples (columns) for D383, D324 and D466 (left to right). Each unique clone is counted once per donor. **B** Principle component analysis (PCA) of *TRBV* gene usage by clone counts per sample for D383. PCA plots are labeled and grouped by lineage, tissue, and subset with confidence ellipses plotted around group mean points using the *factoextra* R package. **C** Barplot showing the mean *TRB* gene usage of CD4^+^ and CD8^+^T cells across 11 individuals with error bars indicating ± standard error. (CD4^+^, *N* = 61; CD8^+^, *N* = 58). t test, ns: *p* > 0.05, *: *p*≤0.05, **: *p*≤0.01, ***: *p*≤0.001, ****: *p*≤0.0001

We further investigated whether there was differential usage of certain *TRBV* genes between subsets and tissues, in the entire sequenced repertoire. *TRBV* gene usage for the three donors where subsets from > 4 sites were obtained reveals certain biases in *TRBV* expression across all donors, with the overall frequencies differing between individuals (Fig. 2A). *TRBV* gene usage for these donors based on lineage, tissue, and subset was assessed by hierarchical clustering and prinicipal component analysis (PCA), revealing distinct clustering by lineage, but not by tissue, and clustering for certain subsets (Fig. 2A,B, Additional File 1: Fig. S2A). Lineage-specific clustering was most apparent by PCA: there was tight clustering of *TRBV* usage for CD4^+^T cells, which was distinct from *TRBV* usage for CD8^+^T cells, which was also more heterogeneous than for CD4^+^T cells (Fig. 2B, Additional File 1: Fig. S2A). Subset-specific clustering patterns for *TRBV* usage were observed for CD8^+^TEMRA which clustered separately from the other subsets (Fig. 2B, Additional File 1: Fig. S2A), consistent with TEMRA cells consisting of a few highly expanded clones.

Similar clustering and heterogeneity patterns for *TRBV* usage for CD4^+^ and CD8^+^T cells and subsets was observed for all 12 donors grouped together (Additional File 1: Fig. S2B), suggesting that *TRBV* usage and clonality are intrinsic features of CD4^+^ and CD8^+^T cell subsets. To address whether specific *TRBV* genes exhibit biased expression in CD4^+^ or CD8^+^ T cells across individuals, we examined TRBV usage for all CD4^+^ and CD8^+^T cells in all donors, identifying significant differences in frequency of 25 different *TRBV* genes between the lineages (greater differences were observed for specific genes *TRBV12.3*, *18*, *27*, *30*, *5.1*, and *6.4* (Fig. 2C), consistent with previous findings [34, 35]. Therefore, the memory T cell repertoire across multiple sites within an individual is selected for specific *TRBV* genes based on CD4^+^ or CD8^+^ lineage, with some skewing by highly expanded subsets.

### Clonal overlap reveals patterns of sharing and relatedness between subsets and sites

**Fig. 3:**
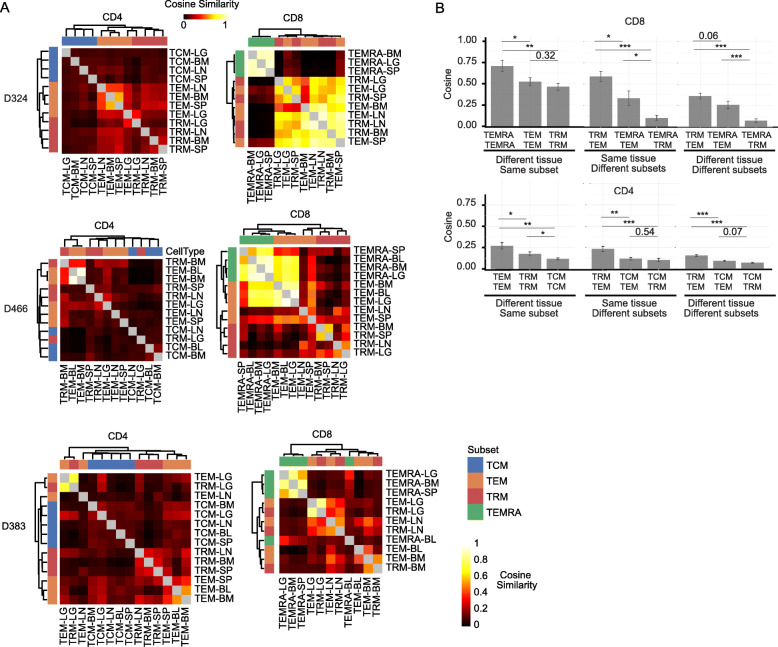
Clonal overlap analysis reveals patterns of dissemination and relatedness between circulating and tissue memory subsets. **A** Cosine similarity between pairwise cell populations of CD4^+^ (left) and CD8^+^ (right) T cell subsets (TCM, TRM, TEM, and TEMRA) from blood and tissues (Lung (LG), bone marrow (BM), lymph node (LN), spleen (SP)) of donor 324 (top), donor 466 (middle), and donor 383 (bottom). Dendrogram created using complete linkage method. Each cell represents the mean of the cosine similarity of replicate samples with the magnitude (min = 0, max = 1) depicted as a heat map. **B** Compiled cosine similarity between subsets and tissues. Average cosine similarly (±SEM) between the same subset in any two sites (BL, BM, SP, LG, or LN) (left), between different subsets within a single site (middle) or between different subsets in different sites (right) for CD8^+^ (upper) and CD4^+^ (lower) T cells

The extent of T cell clonal overlap between tissue sites and subsets could reveal insights into their migration and lineage relationships, respectively. Calculating the cosine similarity [36] between samples within individuals resulted in values ranging from 0 (minimal overlap) to 1 (complete overlap). Heat maps for CD4^+^ and CD8^+^ subsets for each of three donors with > 4 tissues show similarity between samples (Fig. 3A), while compiled cosine similarity values for all donors reveals the overall relatedness of subsets and their migration between sites (Fig. 3B). Overall, CD8^+^T cells had higher cosine similarity (and greater clonal overlap) between sites and subsets compared to CD4^+^T cells (Fig. 3A, B), consistent with their larger clonal expansion (Fig. 1). Between sites, the highest cosine similarity (mean value 0.71, ± 0.07 se) was observed for CD8^+^TEMRA cells in blood, BM, spleen, and lungs of all donors analyzed, indicating that the highly expanded TEMRA clones circulate between tissue sites. CD8^+^TEM cells also exhibited high cosine similarity between sites (mean value 0.53, ± 0.05 se) that was equivalent or greater than for CD8^+^TRM (mean value 0.46 ± 0.04 se), depending on the specific site pairings, though the extent of overlap varied between donors (Fig. 3A, 3B (left)). Similarly, CD4^+^TEM exhibited the highest overlap between sites for CD4^+^subsets (mean value 0.27 ± 0.04 se), consistent with TEM being more circulating, while TCM exhibited the lowest overlap between sites (mean value 0.12 ± 0.01 se) (Fig. 3A, B), consistent with the higher diversity of CD4^+^TCM cells relative to all memory subsets. The extent of overlap for TRM subsets varied between sites; there was higher overlap for CD4^+^TRM between spleen and BM (mean value 0.31 ± 0.03 se), and for CD8^+^TRM between lung and LLN (mean value 0.52 ± 0.06 se) (Fig. 3), suggesting that TRM formation or homeostasis is localized to certain related sites.

We also compared the clonal overlap between different subsets (in the same or different sites) to assess potential lineage relationships. For both CD4^+^ and CD8^+^T cells, TRM and TEM exhibited the greatest overlap within a given site compared to other subsets (mean value ± se: CD4^+^ TRM – TEM 0.23 ± 0.03, CD8^+^ TRM – TEM 0.59 ± 0.06) (Fig. 3B, middle), suggesting that these subsets either derive from a common lineage or interconvert during maintenance. By contrast, CD8^+^TEMRA exhibited minimal overlap with CD8^+^TRM cells within or between sites (mean value ± se: CD8^+^ within sites TEMRA – TRM 0.10 ± 0.03, CD8^+^ between sites TEMRA – TRM 0.07 ± 0.02) (Fig. 3B), and CD4^+^TCM also exhibited minimal overlap with CD4^+^TRM or CD4^+^TEM (mean value ± se: CD4^+^ TCM – TEM 0.12 ± 0.01, CD4^+^ TCM – TRM 0.10 ± 0.02) (Fig. 3B). These findings suggest that CD8^+^TEMRA and CD4^+^TCM are generated along a pathway distinct from the corresponding TEM and TRM cells for CD8^+^ and CD4^+^T cells, respectively. Together, these analyses reveal that circulating and highly expanded subsets (e.g., TEMRA, TEM) are broadly shared across tissue sites, while TRM are less broadly shared between sites, and a potential common clonal origin for certain TEM and TRM subsets.

### Individual clone tracking reveals tissue-specific TRM expansion

**Fig. 4:**
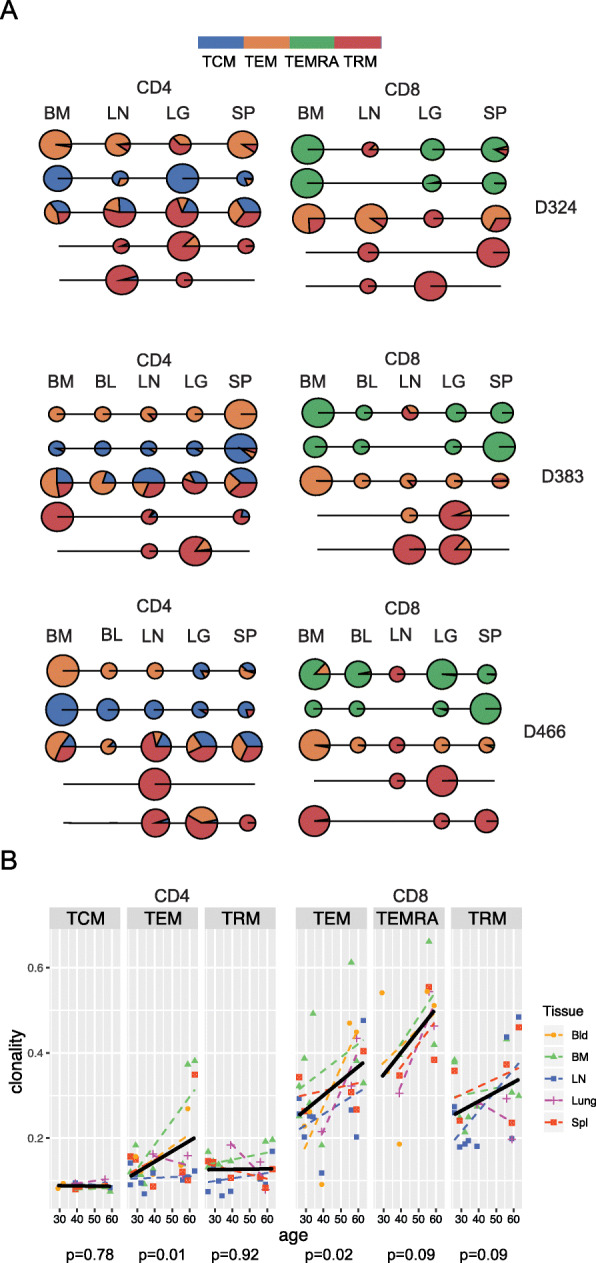
TRM clones exhibit tissue-specific expansions and clonal stability with age. **A** Clone tracking across sites and subsets. Each line-circle plot depicts and individual CD4^+^ or CD8^+^ T cell clone, their subset identity and relative frequency in each site. Each line represents a single clone, circles indicate presence within a site (bone marrow (BM), blood (BL), lung-draining lymph node (LN), lung (LG), or spleen (SP)); the size of the circle is proportional to the total number of copies of the clone in the sample, and colored wedges in the circle indicate what fraction of copies derive from individual subsets (TCM (blue), TEM (orange), TEMRA (green), TRM (red)) from CD4^+^ (*left*) and CD8^+^ (*right*) T cells for D324 (top), D383 (middle) and D466 (bottom). Representative clones shown from the top 500 clones for each donor. **B** Clonality associations with age for each subset. Scatter plots with fitted lines (black) of clonality measurements from Fig. 1 by age from linear regression analysis with associated p values (P) indicated below each plot for CD4^+^ (left) and CD8^+^(right) subsets. Tissue site indicated by shape

To further investigate how individual clones may be shared or segregated within tissues, we tracked the abundance and sharing of large individual clones between subsets and sites for the three donors with 4–5 sites examined, based on similar analysis with B cell clones in tissue sites [37]. Shown in Fig. 4A are the resultant “line-circle plots,” where each line represents one clone spanning 4–5 sites, each circle shows the presence of that clone in a particular site; circles are colored by subset and the size of the circle represents the proportion of the TCR repertoire occupied within each site. We use this analysis to highlight five major patterns of TCR clonal distribution across sites for each lineage that are shared by all three of the most highly sequenced tissue donors. For CD4^+^T cells, certain TEM or TCM clones were distributed similarly across sites, while there were also clones with variable distribution across subsets and sites. Notably, a number of TRM clones were enriched in the lung, BM, or LN, in all donors (Fig. 4A), suggesting site-specific clonal maintenance. For CD8^+^T cells, there were TEMRA or TEM clones distributed across sites, while certain TRM clones were also enriched in single sites such as lung, BM, spleen, or LN (Fig. 4A). This qualitative analysis of clonal distribution showing TRM clones confined to or enriched in certain sites provides strong evidence for tissue-specific maintenance of TRM cells.

### TRM cells maintain clonality over age compared to circulating TEM cells

To further assess TRM maintenance in tissues, we examined potential changes in clonality with age. The clonality of memory T cells in blood is known to increase with age [12], due to the outgrowth of specific clones from persistent stimulation and/or homeostatic maintenance by cytokines and other factors [38]. Consistent with results in blood, we found a significant increase in clonality across all sites with age for both CD4^+^ and CD8^+^TEM cells (Fig. 4B). (TEMRA clonality also exhibited an upward trend with age, which did not achieve significance, possibly due to the already high clonality at younger ages). By contrast, the clonality of CD4^+^TRM and CD4^+^TCM remained strikingly constant in all sites with age and the clonality of CD8^+^TRM also did not exhibit a significant increase with age (Fig. 4B). These results suggest that memory T cells retained in tissues are stably maintained without significant homeostatic expansion or age-associated changes, and may therefore constitute cellular reservoirs for longterm immunological memory.

### Tissue-specific segregation of qualitatively similar clones

**Fig. 5:**
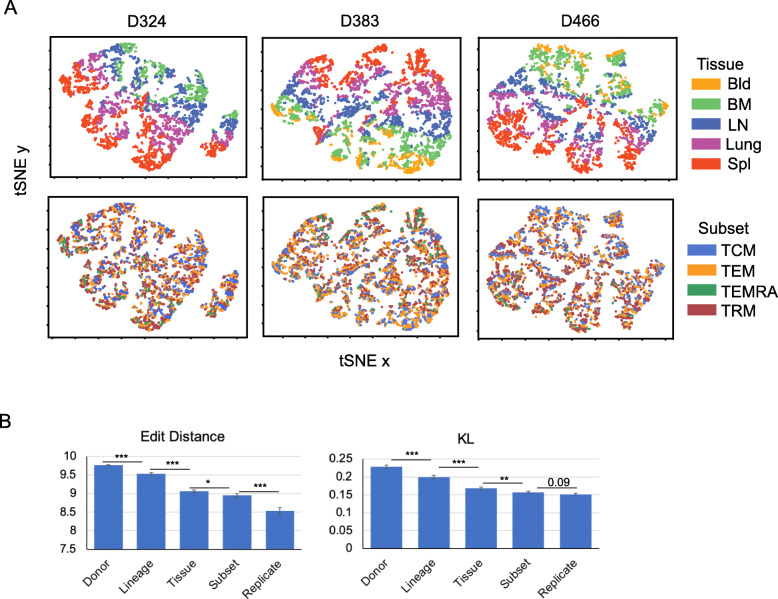
CDR3 protein sequences cluster by tissue site within an individual. **A** Comparing edit distance by tissue and subset. tSNE projections of TCR clones based exclusively on the edit distances between CDR3 amino acid (protein) sequences. Shown are 250 non-overlapping clones that were randomly chosen from each of the three most extensively sampled donors, D324, D383, and D466 (see Methods). Each clone (dot) is colored according to its tissue origin (*upper*) or subset identity (*lower*). TCM, central memory; TEM, effector memory; TEMRA, terminal effector; TRM, resident memory; Bld, blood; BM, bone marrow; LN, lung lymph node; Spl, spleen. **B** Hierarchy of TCR repertoire similarity. Average CDR3 edit distances in amino acid (left) and V gene Kullback-Leibler (KL) divergence (right) are computed for samples by donor, lineage (CD4 or CD8), tissue, subset, and biological replicates from separate DNA aliquots from the same sample (see Methods). A total of 500 clones were randomly chosen from all tissue donor samples for each analysis. Average values and standard errors for each measure were computed. Differences between neighboring samples were computed using a two-sided unpaired t test. * *p* < 0.05; ** *p* < 0.01; *** *p* < 0.001

The preceding analyses have focused on frequency (Figs. 1 and 3), V and J gene usage (Fig. 2), and overlap (Figs. 3 and 4) of individual clones. Next, we took a different approach, by analyzing the amino acid sequence of clones to determine if features that spanned different clones tracked by tissue or subset. In order to do this, we first took each sample and counted each unique clone once and removed clones that overlapped between the different samples from the analysis. We then analyzed CDR3β amino acid sequence similarity using edit distances (Levenshtein [28]) and TRBV sequence similarity using the Kullback-Leibler divergence [27]. These measures are independent of clone size and focused on equivalent numbers of non-overlapping clones for each subset in individual tissue donors (see Methods). CDR3 edit distance matrices visualized in t-SNE plots for all three donors reveal distinct patterns of clustering based on tissue site; clones derived from blood cluster together, next to clusters of clones from BM, adjacent to clusters for LN, lung, and spleen (Fig. 5A, top row). By contrast, tSNE plots of edit distance based on subset revealed intermingling of subsets and no clear pattern of clustering (Fig. 5A, bottom row). The calculated edit distances were not due to differences in CDR3 lengths which were similar between donors, lineages, tissues, and subsets (Additional File 1: Fig. S3A,B), but rather are due to sequence features of the clones.

Compiling the average edit distances for all 9 tissue donors shows the highest edit distances (i.e., most differences) between TCR sequences between individuals, followed by lineages (CD4^+^ or CD8^+^), while the lowest edit distance (i.e., highest similarity) is between sample replicates (Fig. 5B, Additional File 1: Fig. S3C). Between tissues and subsets, the edit distance based on tissue was significantly higher than the magnitude of the edit distance based on TEM, TRM, TEMRA, or TCM subset, consistent with the clustering of similar clones based on tissue, but not subset (Fig. 5B, Additional File 1: Fig. S3C). The same hierachy of donor, lineage, tissue, subset, and replicate was also observed with the Kulback-Leibler (KL) divergence in *TRBV* gene sequences in 500 randomly selected clones (minus overlapping clones) (Fig. 5B) and in 200 randomly selected clones which also includes analysis of Donor 466 which had fewer clones than the other donors (Additional File 1: Fig. S3C). Moreover, similar hierarchies were observed based on calculating Hamming distances [39] (Additional File 1: Figure S4). These results provide evidence for segregation of qualitatively similar clones based on CDR3β amino acid and *TRBV* gene sequences in individual tissue sites, further supporting a role for the tissues in long-term clonal maintenance that is suggested by the stability of TRM clones with age.

## Discussion

Memory T cells are maintained across the body as a record of previous antigen encounters. How specific clones of memory T cells are distributed and maintained in blood and tissues has been challenging to address due to constraints on tissue sampling for humans. Here, we used our validated organ donor tissue resource to investigate the role of subset and tissue in the maintenance of the memory T cell repertoire. We applied HTS to identify the TCR β chain variable region in sorted circulating and tissue resident memory T cell subsets isolated from blood, lymphoid organs, and lung and applied quantitative and qualitative analysis of individual clones across subsets and tissues. Our analysis reveals that quantitative aspects of T cell clonal expansion and sharing between sites are lineage and subset-specific, while the tissue site plays an important role in clonal maintenance. Our results provide a novel assessment of how the memory T cell repertoire is maintained across lineages, subsets, tissues, and age.

Our results identify lineage-specific features of TCR clonal expansion and Vβ usage that are conserved across subsets and sites. T cell lineages differ significantly in the extent of clonal expansion across sites: CD8^+^T cells are more clonally expanded and contain more clones with high copy number relative to CD4^+^T cells, consistent with earlier findings in blood and tissues [8, 12, 13]. Increased clonal expansion by CD8^+^T cells may be due to their increased responses to viruses during an acute response and the continuous surveillance of CD8^+^T cells to persistent viruses. Memory CD4^+^ and CD8^+^T cells also exhibit consistent qualitative differences in *TRVB* usage for all sites, subsets, and individuals, as reported previously for circulating T cells [35], which likely derive from the differences in MHC class II and class I binding motifs, respectively.

In addition to lineage, we also show that the extent of clonal expansion and dissemination across multiple tissue sites are features intrinsic to specific memory T cell subsets. Remarkably, the CD8^+^TEMRA subset in multiple sites and in different individuals consistently showed the highest degree of clonal expansion. A limited number of unique clones (and skewed *TRBV* usage) was represented within this subset—in some individuals, only 10 clones comprised > 80% of the sequenced TEMRA repertoire. As none of the 12 individuals from whom TEMRA clones were sequenced had overt disease, large TEMRA clonal expansions occur within normal T cell homeostasis, and may not be reliable indicators of disease as previously suggested [17]. Extensive clonal expansion of CD8^+^T cells is associated with accumulated responses to acute or persistent viruses, resulting in a narrowing of the TCR repertoire [40], and TEMRA in different tissue sites can be specific for persistence viruses such as CMV [41]. As we observed highly abundant TEMRA clones across sites for both CMV-seropositive and -seronegative individuals (see Table 1), such patterns may reflect exposure to diverse pathogens. The fact that large TEMRA clones disseminated across multiple sites, yet exhibited minimal overlap with TEM, TRM, and TCM subsets, suggests TEMRA cells are generated distinct from memory subsets.

TEM cells also contained expanded clones, a proportion which were shared across sites, albeit to a lesser extent than TEMRA clones. However, a significant proportion of TEM clones were shared with TRM clones both within and across tissues. We propose that this sharing of TEM and TRM clones likely derives from an initial priming event leading to effector expansion and differentiation to circulating and tissue memory T cells. Previous studies in mice showed that a single naïve T cell clone activated in vivo by antigen/adjuvant or virus infection can generate diverse memory subsets (TCM, TEM, TRM, etc.) in multiple sites [42, 43]. Moreover, TCR clonal analysis following skin immunization revealed similar TCR clones in skin TRM and lymphoid memory T cells [42], further indicating that responses originating in tissues can generate widely distributed memory subsets from a common clonal origin. The high overlap for CD8^+^TRM across sites suggests TRM generation from a broadly expanded CD8^+^ effector population that acquires TRM features at local sites.

Our quantitative analysis of TCR clones between and within tissues and within tissue-resident compared to circulating subsets provide several lines of evidence that the tissue plays a significant role in clonal maintenance. Analysis of clonal overlap between sites revealed that TRM clones were less widely shared across sites compared to circulating TEM clones and also exhibited tissue-specific clonal expansions in lung, LN, or BM. Tissue-specific effects on the TCR repertoire were further observed for LN memory CD4^+^ and CD8^+^T cells which had higher TCR diversity, beneficial for antigen-recognition [44], compared to the other sites. These results along with our previous findings that LN memory T cells are more quiescent [33], suggest tissue-specific memory maintenance. Moreover, with age, both CD4^+^ and CD8^+^TRM maintained their clone size while the clonality of circulating memory subsets increased. Together, these quantitative assessments of clonality over sites and age provide evidence for tissues as reservoirs of long-term clonal maintenance.

The edit distance anlaysis reveals a more general role for tissue in the clonal organization of T cell memory. When the amino acid sequences of each unique clone were analyzed for similarity by edit distance measurements, we found clustering of similar TCR sequences by tissue site, but not by subset. These results are further consistent with our single cell transcriptomic profiling of T cells in the same sites analyzed here (lung, BM, LN, blood), showing that all tissue T cells (including TEM and TRM within a site) exhibit tissue-specific gene expression signatures that are distinct from blood [45]. T cells in tissues may be responding to cells and/or factors that promote memory T cell survival, including homeostatic cytokines and MHC molecules with cognate or non-cognate antigen [46, 47], resulting in preferential segregation of TCR clones with a given site.

An understanding of how long-term immunological memories are organized in humans is important for promoting durable protective immunity in vaccines. The question of how T cell immunity in blood can predict protection in sites of infection is particularly relevant in the current pandemic for assessing immunity to infection and vaccines [48, 49]. Our results show that for tissue memory T cells, including TRM and certain TEM clones, blood may be minimally representative of the tissue-enriched populations. The edit distance results further show that blood-derived TCR clones segreagate distinct from clones in tissue sites. A recent study also showed that memory CD8^+^T cell clones in blood were distinct from those circulating through the lymphatics which could transit through tissues [50]. Promoting TRM-mediated protection in mouse models requires site specific, rather than systemic priming [51, 52]. Our findings on the role of the tissue in TCR clonal maintenance in humans indicates that promoting tissue-specific memory responses in humans may likewise require site-specific strategies.

## Conclusions

The human memory T cell repertoire is maintained across multiple sites and different subsets. The quantitative nature of clonal maintenance is a feature of the lineage and subset, while the tissues play a key role in maintaining tissue-adapted clones and serve as reservoirs for stable maintenance of long-term memory responses. Our findings provide a systematic analysis of T cell clones in multiple sites throughout the body.

These data can can serve as a new reference for defining tissue-based T cell immune responses to infection and vaccination and as a comparator for immune repertoires in diseases such as autoimmunity and cancer.

## Supplementary Information

**Additional File 1: Supplementary methods, supplementary tables (S1-S2), and supplementary figures (S1-S3). Table S1.** Number of replicates, copies, unique sequences and clones identified for each individual donor used in this study. **Table S2.** DNA concentration and number of unique clones identified for each T cell sample. **Figure S1.** Gating strategy for T cell subset isolation and workflow for TCR sequencing. **Figure S2.** Principal component analysis (PCA) of *TRBV* gene usage of individual donors and the compiled dataset. **Figure S3.** Tissue segregation of TCR clones across sites.

## Data Availability

The data generated for this study have been uploaded in an Adaptive Immune Receptor Repertoire (AIRR)-compliant manner to SRA/GenBank, BioProject PRJNA638966 [53], available through the following link: https://www.ncbi.nlm.nih.gov/bioproject/PRJNA638966/

## References

[1] Szabo PA, Miron M, Farber DL (2019). Location, location, location: tissue resident memory T cells in mice and humans. Sci Immunol.

[2] Mackay LK, Kallies A (2017). Transcriptional regulation of tissue-resident lymphocytes. Trends Immunol.

[3] Sallusto F, Lenig D, Forster R, Lipp M, Lanzavecchia A (1999). Two subsets of memory T lymphocytes with distinct homing potentials and effector functions [see comments]. Nature.

[4] Boddupalli CS, Bar N, Kadaveru K, Krauthammer M, Pornputtapong N, Mai Z (2016). Interlesional diversity of T cell receptors in melanoma with immune checkpoints enriched in tissue-resident memory T cells. JCI Insight.

[5] Zheng C, Zheng L, Yoo JK, Guo H, Zhang Y, Guo X, Kang B, Hu R, Huang JY, Zhang Q (2017). Landscape of infiltrating T cells in liver cancer revealed by single-cell sequencing. Cell.

[6] Ganesan AP, Clarke J, Wood O, Garrido-Martin EM, Chee SJ, Mellows T, Samaniego-Castruita D, Singh D, Seumois G, Alzetani A, Woo E, Friedmann PS, King EV, Thomas GJ, Sanchez-Elsner T, Vijayanand P, Ottensmeier CH (2017). Tissue-resident memory features are linked to the magnitude of cytotoxic T cell responses in human lung cancer. Nat Immunol.

[7] Wang T, Wang C, Wu J, He C, Zhang W, Liu J, Zhang R, Lv Y, Li Y, Zeng X, Cao H, Zhang X, Xu X, Huang C, Wang L, Liu X (2017). The different T-cell receptor repertoires in breast cancer tumors, draining lymph nodes, and adjacent tissues. Cancer Immunol Res.

[8] Robins HS, Srivastava SK, Campregher PV, Turtle CJ, Andriesen J, Riddell SR, Carlson CS, Warren EH (2010). Overlap and effective size of the human CD8+ T cell receptor repertoire. Sci Transl Med.

[9] Davis MM, Bjorkman PJ (1988). T-cell antigen receptor genes and T-cell recognition. Nature.

[10] Robins H (2013). Immunosequencing: applications of immune repertoire deep sequencing. Curr Opin Immunol.

[11] Robins HS, Campregher PV, Srivastava SK, Wacher A, Turtle CJ, Kahsai O, Riddell SR, Warren EH, Carlson CS (2009). Comprehensive assessment of T-cell receptor beta-chain diversity in alphabeta T cells. Blood.

[12] Qi Q, Liu Y, Cheng Y, Glanville J, Zhang D, Lee JY, Olshen RA, Weyand CM, Boyd SD, Goronzy JJ (2014). Diversity and clonal selection in the human T-cell repertoire. Proc Natl Acad Sci U S A.

[13] Thome JJ, Yudanin N, Ohmura Y, Kubota M, Grinshpun B, Sathaliyawala T, Kato T, Lerner H, Shen Y, Farber DL (2014). Spatial map of human T cell compartmentalization and maintenance over decades of life. Cell.

[14] Soto C, Bombardi RG, Kozhevnikov M, Sinkovits RS, Chen EC, Branchizio A, Kose N, Day SB, Pilkinton M, Gujral M, Mallal S, Crowe JE (2020). High frequency of shared clonotypes in human t cell receptor repertoires. Cell Rep.

[15] Page DB, Yuan J, Redmond D, Wen YH, Durack JC, Emerson R, Solomon S, Dong Z, Wong P, Comstock C, Diab A, Sung J, Maybody M, Morris E, Brogi E, Morrow M, Sacchini V, Elemento O, Robins H, Patil S, Allison JP, Wolchok JD, Hudis C, Norton L, McArthur HL (2016). Deep sequencing of T-cell receptor DNA as a biomarker of clonally expanded TILs in breast cancer after immunotherapy. Cancer Immunol Res.

[16] Matos TR, O'Malley JT, Lowry EL, Hamm D, Kirsch IR, Robins HS, Kupper TS, Krueger JG, Clark RA (2017). Clinically resolved psoriatic lesions contain psoriasis-specific IL-17-producing alphabeta T cell clones. J Clin Invest.

[17] Gate D, Saligrama N, Leventhal O, Yang AC, Unger MS, Middeldorp J, Chen K, Lehallier B, Channappa D, De Los Santos MB (2020). Clonally expanded CD8 T cells patrol the cerebrospinal fluid in Alzheimer’s disease. Nature.

[18] Carpenter DJ, Granot T, Matsuoka N, Senda T, Kumar BV, Thome JJC, Gordon CL, Miron M, Weiner J, Connors T, Lerner H, Friedman A, Kato T, Griesemer AD, Farber DL (2018). Human immunology studies using organ donors: impact of clinical variations on immune parameters in tissues and circulation. Am J Transplant.

[19] Dogra P, Rancan C, Ma W, Toth M, Senda T, Carpenter DJ, Kubota M, Matsumoto R, Thapa P, Szabo PA, Li Poon MM, Li J, Arakawa-Hoyt J, Shen Y, Fong L, Lanier LL, Farber DL (2020). Tissue determinants of human NK cell development, function, and residence. Cell.

[20] Granot T, Senda T, Carpenter DJ, Matsuoka N, Weiner J, Gordon CL, Miron M, Kumar BV, Griesemer A, Ho SH, Lerner H, Thome JJC, Connors T, Reizis B, Farber DL (2017). Dendritic cells display subset and tissue-specific maturation dynamics over human life. Immunity.

[21] Sathaliyawala T, Kubota M, Yudanin N, Turner D, Camp P, Thome JJ, Bickham KL, Lerner H, Goldstein M, Sykes M (2013). Distribution and compartmentalization of human circulating and tissue-resident memory T cell subsets. Immunity.

[22] van Dongen JJ, Langerak AW, Bruggemann M, Evans PA, Hummel M, Lavender FL, Delabesse E, Davi F, Schuuring E, Garcia-Sanz R (2003). Design and standardization of PCR primers and protocols for detection of clonal immunoglobulin and T-cell receptor gene recombinations in suspect lymphoproliferations: report of the BIOMED-2 concerted action BMH4-CT98-3936. Leukemia.

[23] Vander Heiden JA, Yaari G, Uduman M, Stern JN, O'Connor KC, Hafler DA, Vigneault F, Kleinstein SH (2014). pRESTO: a toolkit for processing high-throughput sequencing raw reads of lymphocyte receptor repertoires. Bioinformatics.

[24] Ye J, Ma N, Madden TL, Ostell JM (2013). IgBLAST: an immunoglobulin variable domain sequence analysis tool. Nucleic Acids Res.

[25] Rosenfeld AM, Meng W, Luning Prak ET, Hershberg U (2017). ImmuneDB: a system for the analysis and exploration of high-throughput adaptive immune receptor sequencing data. Bioinformatics.

[26] Rosenfeld AM, Meng W, Luning Prak ET, Hershberg U (2018). ImmuneDB, a novel tool for the analysis, storage, and dissemination of immune repertoire sequencing data. Front Immunol.

[27] Morris H, DeWolf S, Robins H, Sprangers B, LoCascio SA, Shonts BA, Kawai T, Wong W, Yang S, Zuber J (2015). Tracking donor-reactive T cells: Evidence for clonal deletion in tolerant kidney transplant patients. Sci Transl Med.

[28] Levenshtein VI (1966). Binary codes capable of correcting deletions, insertions, and reversals. Soviet Physics Doklady.

[29] Kullback S, Leibler RA (1951). On information and sufficiency. Ann Math Stat.

[30] van der Matten L, Hinton G (2008). Visulazing data using t-SNEJ. Mach Learn Res.

[31] Kumar BV, Ma W, Miron M, Granot T, Guyer RS, Carpenter DJ, Senda T, Sun X, Ho SH, Lerner H, Friedman AL, Shen Y, Farber DL (2017). Human tissue-resident memory T cells are defined by core transcriptional and functional signatures in lymphoid and mucosal sites. Cell Rep.

[32] Thome JJ, Grinshpun B, Kumar BV, Kubota M, Ohmura Y, Lerner H, Sempowski GD, Shen Y, Farber DL (2016). Longterm maintenance of human naive T cells through in situ homeostasis in lymphoid tissue sites. Sci Immunol.

[33] Miron M, Kumar BV, Meng W, Granot T, Carpenter DJ, Senda T, Chen D, Rosenfeld AM, Zhang B, Lerner H, Friedman AL, Hershberg U, Shen Y, Rahman A, Luning Prak ET, Farber DL (2018). Human lymph nodes maintain TCF-1(hi) memory T cells with high functional potential and clonal diversity throughout life. J Immunol.

[34] Davey MP, Meyer MM, Munkirs DD, Babcock D, Braun MP, Hayden JB, Bakke AC (1991). T-cell receptor variable beta genes show differential expression in CD4 and CD8 T cells. Hum Immunol.

[35] Emerson R, Sherwood A, Desmarais C, Malhotra S, Phippard D, Robins H (2013). Estimating the ratio of CD4+ to CD8+ T cells using high-throughput sequence data. J Immunol Methods.

[36] Rosenfeld AM, Meng W, Chen DY, Zhang B, Granot T, Farber DL, Hershberg U, Luning Prak ET (2018). Computational evaluation of B-cell clone sizes in bulk populations. Front Immunol.

[37] Meng W, Zhang B, Schwartz GW, Rosenfeld AM, Ren D, Thome JJC, Carpenter DJ, Matsuoka N, Lerner H, Friedman AL, Granot T, Farber DL, Shlomchik MJ, Hershberg U, Luning Prak ET (2017). An atlas of B-cell clonal distribution in the human body. Nat Biotechnol.

[38] Surh CD, Sprent J (2008). Homeostasis of naive and memory T cells. Immunity.

[39] Hamming RW (1950). Error detecting and error correcting codes. Bell Syst Tech J.

[40] Blackman MA, Woodland DL (2011). The narrowing of the CD8 T cell repertoire in old age. Curr Opin Immunol.

[41] Gordon CL, Miron M, Thome JJ, Matsuoka N, Weiner J, Rak MA, Igarashi S, Granot T, Lerner H, Goodrum F, Farber DL (2017). Tissue reservoirs of antiviral T cell immunity in persistent human CMV infection. J Exp Med.

[42] Gaide O, Emerson RO, Jiang X, Gulati N, Nizza S, Desmarais C, Robins H, Krueger JG, Clark RA, Kupper TS (2015). Common clonal origin of central and resident memory T cells following skin immunization. Nat Med.

[43] Masopust D, Vezys V, Usherwood EJ, Cauley LS, Olson S, Marzo AL, Ward RL, Woodland DL, Lefrancois L (2004). Activated primary and memory CD8 T cells migrate to nonlymphoid tissues regardless of site of activation or tissue of origin. J Immunol.

[44] Wang GC, Dash P, McCullers JA, Doherty PC, Thomas PG (2012). T cell receptor alphabeta diversity inversely correlates with pathogen-specific antibody levels in human cytomegalovirus infection. Sci Transl Med.

[45] Szabo PA, Levitin HM, Miron M, Snyder ME, Senda T, Yuan J, Cheng YL, Bush EC, Dogra P, Thapa P, Farber DL, Sims PA (2019). Single-cell transcriptomics of human T cells reveals tissue and activation signatures in health and disease. Nat Commun.

[46] Purton JF, Tan JT, Rubinstein MP, Kim DM, Sprent J, Surh CD (2007). Antiviral CD4+ memory T cells are IL-15 dependent. J Exp Med.

[47] Surh CD, Boyman O, Purton JF, Sprent J (2006). Homeostasis of memory T cells. Immunol Rev.

[48] Grifoni A, Weiskopf D, Ramirez SI, Mateus J, Dan JM, Moderbacher CR, Rawlings SA, Sutherland A, Premkumar L, Jadi RS, Marrama D, de Silva AM, Frazier A, Carlin AF, Greenbaum JA, Peters B, Krammer F, Smith DM, Crotty S, Sette A (2020). Targets of T cell responses to SARS-CoV-2 coronavirus in humans with COVID-19 disease and unexposed individuals. Cell.

[49] Grigoryan L, Pulendran B (2020). The immunology of SARS-CoV-2 infections and vaccines. Semin Immunol.

[50] Buggert M, Vella LA, Nguyen S, Wu VH, Chen Z, Sekine T, Perez-Potti A, Maldini CR, Manne S, Darko S, Ransier A, Kuri-Cervantes L, Japp AS, Brody IB, Ivarsson MA, Gorin JB, Rivera-Ballesteros O, Hertwig L, Antel JP, Johnson ME, Okoye A, Picker L, Vahedi G, Sparrelid E, Llewellyn-Lacey S, Gostick E, Sandberg JK, Björkström N, Bar-Or A, Dori Y, Naji A, Canaday DH, Laufer TM, Wells AD, Price DA, Frank I, Douek DC, Wherry EJ, Itkin MG, Betts MR (2020). The identity of human tissue-emigrant CD8(+) T cells. Cell.

[51] Zens KD, Chen J-K, Farber DL (2016). Vaccine-generated lung tissue-resident memory T cells provide heterosubtypic protection to influenza infection. J Clin Invest Insight.

[52] Allen AC, Wilk MM, Misiak A, Borkner L, Murphy D, Mills KHG (2018). Sustained protective immunity against Bordetella pertussis nasal colonization by intranasal immunization with a vaccine-adjuvant combination that induces IL-17-secreting TRM cells. Mucosal Immunol.

[53] Miron M, Meng W, Rosenfeld AM, Dvorkin S, Poon MML, Lam N, Kumar BV, Louzoun Y, Luning Prak ET, Farber DL: Maintenance of the human memory T cell repertoire across subsets and tissues. PRJNA638966 NCBI BioProject*.* https://www.ncbi.nlm.nih.gov/bioproject/PRJNA638966/ (2021). 10.1186/s13073-021-00918-7 PMC8204429 34127056

