# Supplementary material for: Maintenance of the human memory T cell repertoire by subset and tissue site
Source: Genome Med. 2021 Jun 14;13:100. doi: 10.1186/s13073-021-00918-7 (PMC8204429; doi:10.1186/s13073-021-00918-7)
Supplement: Supplementary file 1 — Additional File 1: Supplementary methods, supplementary tables (S1-S2), and supplementary figures (S1-S3). Table S1. Number of replicates, copies, unique sequences and clones identified for each individual donor used in this study. Table S2. DNA concentration and number of unique clones identified for each T cell sample. Figure S1. Gating strategy for T cell subset isolation and workflow for TCR sequencing. Figure S2. Principal component analysis (PCA) of TRBV gene usage of individual donors and the compiled dataset. Figure S3. Tissue segregation of TCR clones across sites. [file 13073_2021_918_MOESM1_ESM.docx]

**Additional file 1:**

**Supplementary methods**

# Table S1: Number of replicates, copies, unique sequences and clones identified for each individual donor used in this study.

# Table S2: DNA concentration and number of unique clones identified for each T cell sample.

**Figure S1:** Gating strategy for T-cell subset isolation and workflow for TCR sequencing.

**Figure S2:** Principal component analysis (PCA) of *TRBV* gene usage of individual donors and the compiled dataset.

**Figure S3:** Tissue segregation of TCR clones across sites.

**Supplementary methods**

TCR read counting and clone mapping

Raw reads were pre-processed using pRESTO [23] v0.5.10 and then annotated using IgBLAST’s igblastn command v1.17.0 [24] as shown in **Code 1**. For IgBLAST, the IMGT human TRBV and TRBJ reference databases from October 24, 2019 were used. Low quality sequences were removed if their average shred quality score was less than 30, stretches of bases on each end of all reads that were of low average quality were removed, short sequences (100 bases or fewer) were discarded, and individual bases with a phred low quality score of less than 30 were replaced with an N.  Finally, any sequences with more than 10 such Ns were removed. IgBLAST was then run on the resulting filtered sequences producing AIRR-compliant output files (See Code 1 below).

**Code 1**

PairSeq.py -1 *R1*.fastq -2 *R2*.fastq

AssemblePairs.py align -1 *R1*_pair-pass.fastq \

 -2 *R2*_pair-pass.fastq \

 --coord illumina

FilterSeq.py quality   -s *assemble-pass.fastq

FilterSeq.py trimqual  -s *quality-pass.fastq -q 30 --win 20

FilterSeq.py length    -s *trimqual-pass.fastq -n 100

FilterSeq.py maskqual  -s *length-pass.fastq -q 30

FilterSeq.py missing   -s *maskqual-pass.fastq -n 10

igblastn \
 -germline_db_V IMGT_TRBV.fasta \
 -germline_db_J IMGT_TRBJ.fasta \
 -outfmt 19 \
 -num_threads 12 \
 -domain_system imgt \
 -ig_seqtype TCR \
 -auxiliary_data ${IGDATA}/optional_file/human_gl.aux \
 -organism human

AIRR-compliant output files were then imported into ImmuneDB v0.29.9 were then imported into ImmuneDB v0.29.9 [25, 26] using the *immunedb_import* function [25, 26] (see **Code 2** below). We defined clonally related sequences as those with identical *TRBV* and *TRBJ* gene segments and CDR3 amino acid sequences. We required that a unique sequence be detected at least twice (within an individual) in order to be designated a clone to reduce over estimation of clones due to sequencing errors.

**Code 2**

immunedb_import configs/${DB_NAME}.json airr \

IMGT_TRBV.gapped.fasta \

IMGT_TRBJ.fasta \

.

immunedb_collapse configs/${DB_NAME}.json

immunedb_clones configs/${DB_NAME}.json cluster --min-similarity 1

immunedb_clone_stats configs/${DB_NAME}.json

immunedb_sample_stats configs/${DB_NAME}.json

# Table S1: Number of replicates, copies, unique sequences and clones identified for each individual donor used in this study.

| subject | replicates | copies | uniques | clones |
| --- | --- | --- | --- | --- |
| D229 | 17 | 4306646 | 610103 | 27088 |
| D233 | 46 | 6698069 | 1434078 | 70868 |
| D255 | 46 | 8630600 | 1560033 | 44565 |
| D280 | 30 | 3888316 | 705687 | 56976 |
| D287 | 39 | 9545330 | 1681304 | 78112 |
| D299 | 44 | 15604407 | 2639281 | 81051 |
| D324 | 72 | 13592406 | 2423785 | 92112 |
| D383 | 87 | 8240334 | 2023931 | 106308 |
| D466 | 89 | 7117300 | 1680119 | 79552 |
| HD1 | 16 | 2758901 | 385721 | 26001 |
| HD2 | 16 | 2670388 | 476032 | 30941 |
| HD3 | 14 | 1715048 | 291104 | 27076 |
| Total | 516 | 84767745 | 15911178 | 720650 |

# Table S2: DNA concentration and number of unique clones identified for each T cell sample (replicates 1 and 2 only).

The number of T-cell clones identified by TCR sequencing for individual samples. Each sample name indicates the donor number, anatomical site, lineage, subset, and replicate number of each sample respectively (example: D229_BM_CD4_TEM_1). HD = Blood Donor.

| Sample Name | DNA (ng) | Clones |
| --- | --- | --- |
| D229-BM-CD4-TEM-1 | 23 | 3,215 |
| D229-BM-CD4-TEM-2 | 23 | 3,016 |
| D229-BM-CD4-TRM-1 | 11 | 2,061 |
| D229-BM-CD4-TRM-2 | 11 | 2,020 |
| D229-BM-CD8-TEM-1 | 23 | 1,424 |
| D229-BM-CD8-TEM-2 | 23 | 1,498 |
| D229-BM-CD8-TRM-1 | 23 | 2,073 |
| D229-BM-CD8-TRM-2 | 23 | 1,926 |
| D229-LN-CD4-TEM-1 | 23 | 3,704 |
| D229-LN-CD4-TEM-2 | 23 | 5,736 |
| D229-LN-CD4-TRM-1 | 23 | 3,386 |
| D229-LN-CD4-TRM-2 | 23 | 5,036 |
| D229-LN-CD8-TEM-1 | 23 | 2,091 |
| D229-LN-CD8-TEM-2 | 23 | 2,019 |
| D229-LN-CD8-TRM-1 | 23 | 1,989 |
| D229-LN-CD8-TRM-2 | 23 | 1,815 |
| D233-BM-CD4-TEM-1 | 67 | 4,571 |
| D233-BM-CD4-TEM-2 | 67 | 3,902 |
| D233-BM-CD4-TRM-1 | 67 | 4,052 |
| D233-BM-CD4-TRM-2 | 67 | 3,182 |
| D233-BM-CD8-TEM-1 | 67 | 1,215 |
| D233-BM-CD8-TEM-2 | 67 | 1,305 |
| D233-BM-CD8-TRM-1 | 67 | 1,173 |
| D233-BM-CD8-TRM-2 | 67 | 1,156 |
| D233-LN-CD4-TEM-1 | 67 | 6,381 |
| D233-LN-CD4-TEM-2 | 67 | 5,842 |
| D233-LN-CD4-TRM-1 | 67 | 4,241 |
| D233-LN-CD4-TRM-2 | 67 | 5,288 |
| D233-LN-CD8-TEM-1 | 67 | 1,749 |
| D233-LN-CD8-TEM-2 | 67 | 1,734 |
| D233-LN-CD8-TRM-1 | 67 | 2,111 |
| D233-LN-CD8-TRM-2 | 67 | 2,057 |
| D233-Spl-CD4-TEM-1 | 67 | 2,591 |
| D233-Spl-CD4-TEM-2 | 67 | 5,136 |
| D233-Spl-CD4-TRM-1 | 67 | 4,457 |
| D233-Spl-CD4-TRM-2 | 67 | 4,936 |
| D233-Spl-CD8-TEM-1 | 67 | 1,661 |
| D233-Spl-CD8-TEM-2 | 67 | 1,829 |
| D233-Spl-CD8-TRM-1 | 67 | 753 |
| D233-Spl-CD8-TRM-2 | 67 | 2,386 |
| D255-BM-CD4-TEM-1 | 46 | 745 |
| D255-BM-CD4-TEM-2 | 46 | 1,270 |
| D255-BM-CD4-TRM-1 | 46 | 2,170 |
| D255-BM-CD4-TRM-2 | 46 | 2,581 |
| D255-BM-CD8-TEM-1 | 46 | 237 |
| D255-BM-CD8-TEM-2 | 46 | 718 |
| D255-BM-CD8-TRM-1 | 46 | 865 |
| D255-BM-CD8-TRM-2 | 46 | 1,001 |
| D255-LN-CD4-TEM-1 | 46 | 3,262 |
| D255-LN-CD4-TEM-2 | 46 | 3,589 |
| D255-LN-CD4-TRM-1 | 46 | 4,044 |
| D255-LN-CD4-TRM-2 | 46 | 4,807 |
| D255-LN-CD8-TEM-1 | 46 | 1,343 |
| D255-LN-CD8-TEM-2 | 46 | 1,695 |
| D255-LN-CD8-TRM-1 | 46 | 875 |
| D255-LN-CD8-TRM-2 | 46 | 1,022 |
| D255-Spl-CD4-TEM-1 | 46 | 1,045 |
| D255-Spl-CD4-TEM-2 | 46 | 1,248 |
| D255-Spl-CD4-TRM-1 | 46 | 3,321 |
| D255-Spl-CD4-TRM-2 | 46 | 7,542 |
| D255-Spl-CD8-TEM-1 | 46 | 582 |
| D255-Spl-CD8-TEM-2 | 46 | 647 |
| D255-Spl-CD8-TRM-1 | 46 | 912 |
| D255-Spl-CD8-TRM-2 | 46 | 1,027 |
| D280-BM-CD4-TEM-1 | 26 | 5,250 |
| D280-BM-CD4-TEM-2 | 26 | 5,826 |
| D280-BM-CD4-TRM-1 | 26 | 986 |
| D280-BM-CD4-TRM-2 | 26 | 734 |
| D280-BM-CD8-TEM-1 | 26 | 830 |
| D280-BM-CD8-TEM-2 | 26 | 1,008 |
| D280-BM-CD8-TRM-1 | 26 | 1,102 |
| D280-BM-CD8-TRM-2 | 26 | 1,012 |
| D280-LN-CD4-TEM-1 | 26 | 1,888 |
| D280-LN-CD4-TEM-2 | 26 | 1,436 |
| D280-LN-CD4-TRM-1 | 26 | 2,806 |
| D280-LN-CD4-TRM-2 | 26 | 3,026 |
| D280-LN-CD8-TEM-1 | 26 | 1,469 |
| D280-LN-CD8-TEM-2 | 26 | 1,256 |
| D280-LN-CD8-TRM-1 | 26 | 888 |
| D280-LN-CD8-TRM-2 | 26 | 1,362 |
| D287-BM-CD4-TEM-1 | 59 | 6,696 |
| D287-BM-CD4-TEM-2 | 59 | 6,952 |
| D287-BM-CD4-TRM-1 | 50 | 3,853 |
| D287-BM-CD4-TRM-2 | 50 | 4,216 |
| D287-BM-CD8-TEM-1 | 69 | 1,262 |
| D287-BM-CD8-TEM-2 | 69 | 1,426 |
| D287-BM-CD8-TRM-1 | 35 | 2,300 |
| D287-BM-CD8-TRM-2 | 35 | 2,032 |
| D287-LN-CD4-TEM-1 | 63 | 8,236 |
| D287-LN-CD4-TEM-2 | 63 | 7,750 |
| D287-LN-CD4-TRM-1 | 73 | 7,890 |
| D287-LN-CD4-TRM-2 | 73 | 8,533 |
| D287-LN-CD8-TEM-1 | 55 | 3,636 |
| D287-LN-CD8-TEM-2 | 55 | 3,761 |
| D287-LN-CD8-TRM-1 | 14 | 1,363 |
| D287-LN-CD8-TRM-2 | 14 | 1,558 |
| D299-BM-CD4-TEM-1 | 41 | 1,959 |
| D299-BM-CD4-TEM-2 | 41 | 1,930 |
| D299-BM-CD4-TRM-1 | 41 | 2,692 |
| D299-BM-CD4-TRM-2 | 41 | 2,767 |
| D299-BM-CD8-TEM-1 | 41 | 660 |
| D299-BM-CD8-TEM-2 | 41 | 553 |
| D299-BM-CD8-TRM-1 | 41 | 1,048 |
| D299-BM-CD8-TRM-2 | 41 | 1,087 |
| D299-LN-CD4-TEM-1 | 41 | 2,404 |
| D299-LN-CD4-TEM-2 | 41 | 2,341 |
| D299-LN-CD4-TRM-1 | 41 | 1,697 |
| D299-LN-CD4-TRM-2 | 41 | 1,596 |
| D299-LN-CD8-TEM-1 | 41 | 1,453 |
| D299-LN-CD8-TEM-2 | 41 | 1,484 |
| D299-LN-CD8-TRM-1 | 41 | 2,052 |
| D299-LN-CD8-TRM-2 | 41 | 2,188 |
| D299-Spl-CD4-TEM-1 | 41 | 1,982 |
| D299-Spl-CD4-TEM-2 | 41 | 1,978 |
| D299-Spl-CD4-TRM-1 | 41 | 2,408 |
| D299-Spl-CD4-TRM-2 | 41 | 2,459 |
| D299-Spl-CD8-TEM-1 | 41 | 881 |
| D299-Spl-CD8-TEM-2 | 41 | 870 |
| D299-Spl-CD8-TRM-1 | 41 | 1,327 |
| D299-Spl-CD8-TRM-2 | 41 | 1,261 |
| D324-BM-CD4-TCM-1 | 15 | 3,168 |
| D324-BM-CD4-TCM-2 | 15 | 3,233 |
| D324-BM-CD4-TEM-1 | 15 | 2,474 |
| D324-BM-CD4-TEM-2 | 15 | 2,373 |
| D324-BM-CD4-TRM-1 | 15 | 2,869 |
| D324-BM-CD8-TEMRA-1 | 12 | 123 |
| D324-BM-CD8-TEMRA-2 | 12 | 108 |
| D324-BM-CD8-TEM-1 | 15 | 273 |
| D324-BM-CD8-TEM-2 | 15 | 866 |
| D324-BM-CD8-TRM-1 | 11 | 555 |
| D324-BM-CD8-TRM-2 | 11 | 678 |
| D324-LN-CD4-TCM-1 | 15 | 3,667 |
| D324-LN-CD4-TCM-2 | 15 | 3,573 |
| D324-LN-CD4-TEM-1 | 15 | 3,206 |
| D324-LN-CD4-TEM-2 | 15 | 3,089 |
| D324-LN-CD4-TRM-1 | 15 | 3,395 |
| D324-LN-CD4-TRM-2 | 15 | 3,219 |
| D324-LN-CD8-TEM-1 | 13 | 1,343 |
| D324-LN-CD8-TEM-2 | 13 | 1,324 |
| D324-LN-CD8-TRM-1 | 15 | 773 |
| D324-LN-CD8-TRM-2 | 15 | 794 |
| D324-LG-CD4-TCM-1 | 15 | 1,962 |
| D324-LG-CD4-TCM-2 | 15 | 2,242 |
| D324-LG-CD4-TEM-1 | 15 | 2,283 |
| D324-LG-CD4-TEM-2 | 15 | 2,101 |
| D324-LG-CD4-TRM-1 | 15 | 2,260 |
| D324-LG-CD4-TRM-2 | 15 | 2,095 |
| D324-LG-CD8-TEMRA-1 | 15 | 298 |
| D324-LG-CD8-TEMRA-2 | 15 | 322 |
| D324-LG-CD8-TEM-1 | 15 | 811 |
| D324-LG-CD8-TEM-2 | 15 | 1,168 |
| D324-LG-CD8-TRM-1 | 15 | 1,153 |
| D324-LG-CD8-TRM-2 | 15 | 1,103 |
| D324-Spl-CD4-TCM-1 | 15 | 4,024 |
| D324-Spl-CD4-TCM-2 | 15 | 3,728 |
| D324-Spl-CD4-TEM-1 | 15 | 3,228 |
| D324-Spl-CD4-TEM-2 | 15 | 3,112 |
| D324-Spl-CD4-TRM-1 | 15 | 3,601 |
| D324-Spl-CD4-TRM-2 | 15 | 3,570 |
| D324-Spl-CD8-TEMRA-1 | 15 | 420 |
| D324-Spl-CD8-TEMRA-2 | 15 | 270 |
| D324-Spl-CD8-TEM-1 | 15 | 2,310 |
| D324-Spl-CD8-TEM-2 | 15 | 2,125 |
| D324-Spl-CD8-TRM-1 | 15 | 993 |
| D324-Spl-CD8-TRM-2 | 15 | 421 |
| D383-BM-CD4-TCM-1 | 33 | 5,403 |
| D383-BM-CD4-TCM-2 | 33 | 4,299 |
| D383-BM-CD4-TEM-1 | 50 | 4,176 |
| D383-BM-CD4-TEM-2 | 50 | 3,752 |
| D383-BM-CD4-TRM-1 | 20 | 2,972 |
| D383-BM-CD4-TRM-2 | 20 | 3,080 |
| D383-BM-CD8-TEMRA-1 | 2 | 769 |
| D383-BM-CD8-TEMRA-2 | 50 | 723 |
| D383-BM-CD8-TEM-1 | 50 | 907 |
| D383-BM-CD8-TEM-2 | 50 | 1,743 |
| D383-BM-CD8-TRM-1 | 50 | 1,244 |
| D383-BM-CD8-TRM-2 | 50 | 1,111 |
| D383-Bld-CD4-TCM-1 | 50 | 3,860 |
| D383-Bld-CD4-TCM-2 | 50 | 3,912 |
| D383-Bld-CD4-TEM-1 | 50 | 2,326 |
| D383-Bld-CD4-TEM-2 | 50 | 2,446 |
| D383-Bld-CD8-TEMRA-1 | 50 | 324 |
| D383-Bld-CD8-TEMRA-2 | 50 | 223 |
| D383-Bld-CD8-TEM-1 | 50 | 3,209 |
| D383-Bld-CD8-TEM-2 | 50 | 3,318 |
| D383-LN-CD4-TCM-1 | 50 | 6,536 |
| D383-LN-CD4-TCM-2 | 50 | 3,817 |
| D383-LN-CD4-TEM-1 | 50 | 9,314 |
| D383-LN-CD4-TEM-2 | 50 | 8,187 |
| D383-LN-CD4-TRM-1 | 50 | 8,927 |
| D383-LN-CD4-TRM-2 | 50 | 3,017 |
| D383-LN-CD8-TEM-1 | 50 | 4,559 |
| D383-LN-CD8-TEM-2 | 50 | 4,262 |
| D383-LN-CD8-TRM-1 | 50 | 4,249 |
| D383-LN-CD8-TRM-2 | 50 | 2,158 |
| D383-LG-CD4-TCM-1 | 50 | 5,045 |
| D383-LG-CD4-TCM-2 | 50 | 5,200 |
| D383-LG-CD4-TEM-1 | 50 | 3,538 |
| D383-LG-CD4-TEM-2 | 50 | 3,414 |
| D383-LG-CD4-TRM-1 | 50 | 3,771 |
| D383-LG-CD4-TRM-2 | 50 | 1,925 |
| D383-LG-CD8-TEMRA-1 | 50 | 771 |
| D383-LG-CD8-TEMRA-2 | 50 | 659 |
| D383-LG-CD8-TEM-1 | 50 | 6,343 |
| D383-LG-CD8-TEM-2 | 33 | 6,479 |
| D383-LG-CD8-TRM-1 | 50 | 1,804 |
| D383-LG-CD8-TRM-2 | 50 | 1,757 |
| D383-Spl-CD4-TCM-1 | 50 | 5,923 |
| D383-Spl-CD4-TCM-2 | 50 | 5,322 |
| D383-Spl-CD4-TEM-1 | 50 | 6,056 |
| D383-Spl-CD4-TEM-2 | 50 | 4,961 |
| D383-Spl-CD4-TRM-1 | 50 | 5,243 |
| D383-Spl-CD4-TRM-2 | 50 | 5,321 |
| D383-Spl-CD8-TEMRA-1 | 50 | 1,443 |
| D383-Spl-CD8-TEMRA-2 | 50 | 751 |
|  |  |  |
|  |  |  |
| HD1-Bld-CD4-TCM-1 | 65 | 7,252 |
| HD1-Bld-CD4-TCM-2 | 65 | 4,844 |
| HD1-Bld-CD8-TEM-1 | 65 | 1,187 |
| HD1-Bld-CD8-TEM-2 | 65 | 1,199 |
| HD1-Bld-CD8-TEMRA-1 | 65 | 579 |
| HD1-Bld-CD8-TEMRA-2 | 65 | 601 |
| HD1-Bld-CD4-TEM-1 | 65 | 3,952 |
| HD1-Bld-CD4-TEM-2 | 65 | 3,766 |
| HD2-Bld-CD4-TCM-1 | 65 | 6,252 |
| HD2-Bld-CD4-TCM-2 | 65 | 5,882 |
| HD2-Bld-CD4-TEM-1 | 65 | 4,951 |
| HD2-Bld-CD4-TEM-2 | 65 | 3,539 |
| HD2-Bld-CD8-TEMRA-1 | 65 | 2,436 |
| HD2-Bld-CD8-TEMRA-2 | 65 | 1,876 |
| HD2-Bld-CD8-TEM-1 | 65 | 2,775 |
| HD2-Bld-CD8-TEM-2 | 65 | 2,742 |
| HD3-Bld-CD4-TCM-1 | 65 | 4,640 |
| HD3-Bld-CD4-TCM-2 | 65 | 4,658 |
| HD3-Bld-CD4-TEM-1 | 65 | 5,211 |
| HD3-Bld-CD4-TEM-2 | 65 | 4,712 |
| HD3-Bld-CD8-TEMRA-1 | 65 | 586 |
| HD3-Bld-CD8-TEMRA-2 | 65 | 552 |
| HD3-Bld-CD8-TEM-1 | 65 | 1,291 |
| HD3-Bld-CD8-TEM-2 | 65 | 1,479 |
| D466-BM-CD4-TCM-1 | 25 | 3,402 |
| D466-BM-CD4-TCM-2 | 25 | 3,372 |
| D466-BM-CD4-TEM-1 | 25 | 915 |
| D466-BM-CD4-TEM-2 | 25 | 942 |
| D466-BM-CD4-TRM-1 | 25 | 2,544 |
| D466-BM-CD8-TEMRA-1 | 25 | 246 |
| D466-BM-CD8-TEMRA-2 | 25 | 777 |
| D466-BM-CD8-TEM-1 | 25 | 1,395 |
| D466-BM-CD8-TEM-2 | 25 | 535 |
| D466-BM-CD8-TRM-1 | 25 | 1,167 |
| D466-BM-CD8-TRM-2 | 25 | 1,323 |
| D466-LN-CD4-TCM-1 | 25 | 3,153 |
| D466-LN-CD4-TCM-2 | 25 | 3,166 |
| D466-LN-CD4-TEM-1 | 25 | 2,766 |
| D466-LN-CD4-TEM-2 | 25 | 2,802 |
| D466-LN-CD4-TRM-1 | 25 | 3,161 |
| D466-LN-CD4-TRM-2 | 25 | 2,907 |
| D466-LN-CD8-TEMRA-1 | 25 | 373 |
| D466-LN-CD8-TEM-1 | 25 | 1,131 |
| D466-LN-CD8-TRM-1 | 25 | 1,887 |
| D466-BL-CD4-TCM-1 | 25 | 3,709 |
| D466-BL-CD4-TCM-2 | 25 | 3,503 |
| D466-BL-CD4-TEM-1 | 25 | 2,460 |
| D466-BL-CD4-TRM-1 | 25 | 107 |
| D466-BL-CD8-TEMRA-1 | 25 | 163 |
| D466-BL-CD8-TEMRA-2 | 25 | 156 |
| D466-BL-CD8-TEM-1 | 25 | 797 |
| D466-BL-CD8-TRM-1 | 25 | 452 |
| D466-LG-CD4-TCM-1 | 25 | 1,755 |
| D466-LG-CD4-TCM-2 | 25 | 2,841 |
| D466-LG-CD4-TEM-1 | 25 | 2,499 |
| D466-LG-CD4-TEM-2 | 25 | 2,217 |
| D466-LG-CD4-TRM-1 | 25 | 3,343 |
| D466-LG-CD4-TRM-2 | 25 | 3,430 |
| D466-LG-CD8-TEMRA-1 | 25 | 202 |
| D466-LG-CD8-TEMRA-2 | 25 | 223 |
| D466-LG-CD8-TEM-1 | 25 | 824 |
| D466-LG-CD8-TRM-1 | 25 | 1,470 |
| D466-LG-CD8-TRM-2 | 25 | 1,405 |
| D466-Spl-CD4-TCM-1 | 25 | 2,042 |
| D466-Spl-CD4-TCM-2 | 25 | 2,095 |
| D466-Spl-CD4-TEM-1 | 25 | 3,211 |
| D466-Spl-CD4-TEM-2 | 25 | 3,354 |
| D466-Spl-CD4-TRM-1 | 25 | 3,180 |
| D466-Spl-CD4-TRM-2 | 25 | 3,223 |
| D466-Spl-CD8-TEMRA-1 | 25 | 706 |
| D466-Spl-CD8-TEMRA-2 | 25 | 629 |
| D466-Spl-CD8-TEM-1 | 25 | 1,205 |
| D466-Spl-CD8-TEM-2 | 25 | 681 |
| D466-Spl-CD8-TRM-1 | 25 | 778 |
| D466-Spl-CD8-TRM-2 | 25 | 1,019 |

**Figure S1. Gating strategy for T-cell subset isolation and workflow for TCR sequencing.** A. Representative Flow cytometry of the major T cell subsets for CD4^+^ (left) and CD8^+^ (right) T cells delineated by CD45RA and CCR7 expression into naïve (CD45RA^+^CCR7^+^), TCM (CD45RA^-^CCR7^+^), TEM (CD45RA^-^CCR7^-^), and TEMRA (CD45RA^+^CCR7^-^) cells in 4 different sites from Donor 324. TRM cells were defined as CD69^+^TEM. B. Average subset composition in blood and 4 tissue sites from all donors in this study for CD4^+^ (upper) and CD8^+^ (lower) T cells. C. Gating strategy for isolating T cells populations for TCR sequencing as follows: Lymphocytes were gated by FSC-A and SSC-A, singlets by trigger cell width (not shown), CD3^+^, CD4^+^CD8^-^ or CD8^+^CD4^-^ cells (negative gates not shown). CD4^+^ T-cell subsets were sorted to on the indicated markers and as designated in (A) to isolate central-memory (TCM) (CD45RA^-^ CCR7^+^), TEM (CD45RA^-^ CCR7^-^CD69^-^), and TRM (CD45RA^-^CCR7^-^CD69^+^) cells and CD8^+^T cells were sorted for isolation of TEMRA cells (CD45RA^+^CCR7^-^), TEM (CD45RA^-^ CCR7^-^CD69^-^), and TRM (CD45RA^-^CCR7^-^CD69^+^) cells. D. DNA was extracted from T cells sorted as in (A) and divided into two replicate samples for sequencing. After multiple individual PCRs on each replicate (with primers targeting the *TRB* gene, see methods) and sequencing, the data were processed to identify clones by unique *TRBV* and *TRBJ* gene segment pairing and CDR3 amino acid sequence (see methods).

**Figure S2. Principal component analysis (PCA) of *TRBV* gene usage of individual donors and the compiled dataset. A.** PCA plots of *TRBV* data using frequency values for the TRBV genes shown in Figure 2 for Donor 324 (left column) and Donor 466 (right column) are labeled and grouped by Lineage, Tissue, and Subset with confidence ellipses plotted around group mean points using the factoextra R package. B. PCA plots calculated and grouped as in (A) but with all donors combined. Plots are labeled and grouped by Lineage, Tissue, Subset, and Donor.

**Figure S3. Overlap and tissue segregation of TCR clones across sites.** A. Visualization of edit distances for largest 500 clones by tSNE colored by CDR3 length. B. CDR3 length distribution for in-frame (IF) and out-of-frame (OF) CDR3 sequences divided by group: Lineage/Donor/Tissue/Subset. There is a clear difference in both the average and standard deviation of length between IF and OF rearrangement, but no difference between the sample groups. C. Edit distances and KL as in Figure 5 but for the top 200 clones, now including D466 for which fewer total sequences were obtained. D. Hamming distances between samples from different Donors, Tissues or Subsets, shown for a set of 500 randomly selected non-overlapped clones for CD4 (left) and CD8 T cells (right).
